# Prevalence of Self-Reported Diagnosed Diabetes Among Adults, by County Metropolitan Status and Region, United States, 2019–2022

**DOI:** 10.5888/pcd21.240221

**Published:** 2024-10-17

**Authors:** Stephen Onufrak, Ryan Saelee, Ibrahim Zaganjor, Yoshihisa Miyamoto, Alain K. Koyama, Fang Xu, Meda E. Pavkov, Kai McKeever Bullard, Giuseppina Imperatore

**Affiliations:** 1 Division of Diabetes Translation, National Center for Chronic Disease Prevention and Health Promotion, Centers for Disease Control and Prevention, Atlanta, Georgia

## Abstract

**Introduction:**

Previous research suggests that rural–urban disparities in diabetes mortality, hospitalization, and incidence rates may manifest differently across US regions. However, no studies have examined disparities in diabetes prevalence by metropolitan residence and region.

**Methods:**

We used data from the 2019–2022 National Health Interview Survey to compare diabetes status, socioeconomic characteristics, and weight status among adults in each census region (Northeast, Midwest, South, West) according to county metropolitan status of residence (large central metro, large fringe metro, small/medium metro, and nonmetro). We used χ^2^ tests and logistic regression models to assess the association of metropolitan residence with diabetes prevalence in each region.

**Results:**

Diabetes prevalence ranged from 7.0% in large fringe metro counties in the Northeast to 14.8% in nonmetro counties in the South. Compared with adults from large central metro counties, those from small/medium metro counties had significantly higher odds of diabetes in the Midwest (age-, sex-, and race and ethnicity–adjusted odds ratio [OR] = 1.24; 95% CI, 1.06–1.45) and South (OR = 1.15; 95% CI, 1.02–1.30). Nonmetro residence was also associated with diabetes in the South (OR = 1.62 vs large central metro; 95% CI, 1.43–1.84). After further adjustment for socioeconomic and body weight status, small/medium metro associations with diabetes became nonsignificant, but nonmetro residence in the South remained significantly associated with diabetes (OR = 1.22; 95% CI, 1.07–1.39).

**Conclusion:**

The association of metropolitan residence with diabetes prevalence differs across US regions. These findings can help to guide efforts in areas where diabetes prevention and care resources may be better directed.

**Summary**

**What is already known on this topic?**

Rural–urban disparities in diabetes mortality, hospitalization, and incidence rates may manifest differently across US regions.

**What is added by this report?**

We found that the association of metropolitan residence with diabetes prevalence differs across regions of the US. Diabetes prevalence ranged from 7.0% in large fringe metro counties in the Northeast to 14.8% in nonmetro counties in the South.

**What are the implications for public health practice?**

These findings can help guide efforts in areas where diabetes prevention and care resources may be better directed.

## Introduction

Diabetes is a costly chronic disease that shortens lifespans and leads to substantial illness that negatively affects quality of life. In 2021, approximately 8.5% of the US adult population had diagnosed diabetes, although prevalence varied widely among states and territories, ranging from 14.4% in Puerto Rico to 6.5% in Colorado (1). The substantial geographic variation of prevalence estimates may be driven partly by differences in age, race and ethnicity, and socioeconomic status (2). However, other contextual factors such as access to health care, the built environment, behavioral risk factors such as physical inactivity, and cultural elements such as dietary patterns may further affect diabetes prevalence. Rural areas in the US have higher prevalence of obesity (3), heart disease (4), stroke mortality (5), and chronic disease risk factors such as cigarette smoking (6), physical inactivity (7), and poor nutrition (8). Diabetes mortality rates are also higher in rural counties than urban counties and have declined more slowly than in urban counties in recent decades (9).

Rural areas in the US are diverse in terms of land use, employment, and culture. Previous research suggests that rural–urban disparities in diabetes mortality, hospitalization, and incidence rates may manifest differently across different US regions. For example, urban–rural disparities in diabetes mortality rates appear to be greater in the South census region and lesser in the West region compared with the Northeast and Midwest regions (10). Similar patterns have also been observed in diabetes-related hospitalization rates following an emergency department visit (11). Finally, health care data from the Veterans Administration also suggests higher incidence of type 2 diabetes in the rural South and in higher-density urban environments of the Northeast and West than in other areas of the US (12). Although diabetes prevalence is a function of both diabetes incidence (new cases) and mortality (survival of existing cases), no recent studies have examined how disparities of diabetes prevalence according to urban/rural status may vary according to region. Therefore, the purpose of this study was to examine differences in the association of diabetes prevalence and urban/rural status of residence by region, as well as how demographic and socioeconomic factors and weight status may help to explain any observed disparities.

## Methods

We used data from the 2019–2022 National Health Interview Survey (NHIS), an annual survey of US households and noninstitutional group quarters (eg, college dormitories, group homes) from the 50 states and the District of Columbia (13). The sample is drawn using a geographically clustered design in a manner such that each month’s sample is nationally representative. A sample adult from each household responds to various survey questions regarding health status and behaviors and demographic and socioeconomic characteristics. Most interviews are conducted face-to-face using a computer-aided personal interview, although some interviews are conducted, in part or whole, over the telephone. For 2019–2022, NHIS sample sizes and final response rates for sample adults were 31,997 (59.1%) for 2019; 21,153 (48.9%) for 2020; 29,482 (50.9%) for 2021; and 27,651 (47.7%) for 2022 (13). Participants from the 2019 NHIS who were reinterviewed in 2020 as part of a one-time NHIS longitudinal data collection were only included in the 2019 sample. For the present study, 110,283 participants were included across all years. A total of 725 participants were excluded due to missing data for diabetes status (n = 135), educational attainment (n = 590), sex (n = 9), or a combination of these variables, resulting in a final analytic sample of 109,558.

The primary outcome, diabetes status, was based on self-report of physician diagnosis ascertained with the question, “(Not including gestational diabetes or prediabetes) Has a doctor or other health professional EVER told you that you had diabetes?” The primary predictor variables were region, which was classified according to the US census regions (Northeast, Midwest, South, and West) and metropolitan residence, which was based on the county of residence of the household and serves as a proxy for urban/rural status. Metropolitan residence was classified based on the 6 categories of the 2013 National Center for Health Statistics (NCHS) Urban–Rural Classification Scheme, which are collapsed into 4 categories in NHIS public use data sets: large central metro, large fringe metro, medium and small metro, and nonmetro (includes micropolitan and noncore) (14). Demographic variables were age (18–44 y, 45–64 y, 65–74 y, and ≥75 y), sex (female, male), and race and ethnicity (Hispanic, non-Hispanic [NH] Asian, NH Black, NH White, or NH Other). Socioeconomic status variables were educational attainment (less than high school, high school or equivalent, some college or associate degree, or bachelor’s degree and above) and family income-to-poverty ratio (<100%, 100%–199%, 200%–299%, 300%–399%, 400%–499%, or ≥500%), the ratio of annual family income to the poverty threshold for household size. Because of missing or incomplete data on family income, approximately 23% to 24% of family income-to-poverty ratio values for each survey year were replaced with a single imputation provided by NCHS. Body weight status was based on self-reported height and weight and classified according to body mass index (BMI, kg/m^2^) as underweight or normal weight (<25.0), overweight (25.0–29.9), obese (≥30.0), or missing.

### Statistical analysis

Analysis was conducted using SAS version 9.4 (SAS Institute) with survey procedures to account for sample weights and survey design variables. Significance was set at *P < .*05. Diabetes status, demographic and socioeconomic characteristics, and body weight status were compared within each region according to metropolitan residence using the Rao-Scott F-adjusted χ^2^ test. Odds ratios (ORs) with 95% CIs from logistic regression models were used to assess the association between metropolitan residence and diabetes prevalence within each region. In all models, the interaction between region and metropolitan residence was tested using type 3 analysis of effects F-test, and a SLICE statement was used to perform a partitioned analysis to estimate the effect of metropolitan residence on diabetes prevalence within each region. In addition to region and metropolitan residence, the first model also included age, sex, and race and ethnicity as covariates. The second model additionally included income-to-poverty ratio and educational attainment, and the third model included variables from the second model plus body weight status.

## Results

Age differed significantly by metropolitan residence for all regions, with large central and fringe metro counties containing a younger population compared with nonmetro counties (Table 1a and Table 1b). Race and ethnicity distribution also differed according to metropolitan residence across all regions, with NH White adults constituting most (≥71%) residents of nonmetro areas in every region. For all regions, educational attainment was lower among adults from nonmetro counties compared with those from large metro counties. Income and body weight status also differed by metropolitan residence across all regions, with residents from nonmetro counties having lower incomes and greater prevalence of obesity.

**Table 1a:** Demographic Characteristics of Adults, by Region and County Metropolitan Status, US Northeast and Midwest Regions, National Health Interview Survey, 2019–2022

Characteristic	Northeast (n = 18,461), % (95% CI)				Midwest (n = 24,081), % (95% CI)			
	Large central metro (n = 5,250)	Large fringe metro (n = 6,788)	Small/medium metro (n = 5,005)	Nonmetro (n = 1,418)	Large central metro (n = 5,110)	Large fringe metro (n = 6,005)	Small/medium metro (n = 7,180)	Nonmetro (n = 5,786)
**Age, y**								
18–44	48.0 (45.8–50.2)	43.0 (41.3–44.6)	40.3 (38.3–42.3)	36.6 (33.5–39.7)	50.8 (48.8–52.8)	44.6 (42.8–46.3)	48.4 (45.8–50.9)	37.8 (35.9–39.7)
45–64	31.4 (29.9–32.9)	34.1 (32.9–35.3)	35.1 (33.5–36.7)	36.1 (33.8–38.4)	30.7 (28.9–32.5)	34.2 (32.7–35.7)	30.2 (28.3–32.1)	34.7 (33.3–36.1)
65–74	12.1 (11.1–13.2)	13.3 (12.5–14.1)	14.5 (13.0–16.1)	16.5 (15.3–17.7)	11.7 (10.7–12.7)	12.8 (11.8–13.7)	12.6 (11.8–13.5)	15.4 (14.5–16.4)
≥75	8.5 (7.5–9.4)	9.7 (8.9–10.4)	10.1 (9.3–10.9)	10.7 (9.0–12.5)	6.9 (6.1–7.7)	8.5 (7.5–9.5)	8.8 (7.9–9.7)	12.1 (11.0–13.2)
**Sex**								
Female	51.9 (50.1–53.6)^a^	51.1 (49.6–52.6)^a^	51.8 (50.0–53.6)^a^	51.0 (46.4–55.6)^a^	51.1 (49.6–52.6)^a^	52.0 (50.4–53.5)^a^	51.5 (49.9–53.1)^a^	50.2 (48.2–52.1)^a^
Male	48.1 (46.4–49.9)^a^	48.9 (47.4–50.4)^a^	48.2 (46.4–50.0)^a^	49.0 (44.4–53.6)^a^	48.9 (47.4–50.4)^a^	48.0 (46.5–49.5)^a^	48.5 (46.9–50.1)^a^	49.8 (47.9–51.8)^a^
**Race and ethnicity**								
Hispanic	19.9 (16.3–23.5)	11.1 (8.8–13.4)	8.9 (6.7–11.1)	2.8 (0.1–5.5)	11.5 (8.9–14.1)	6.8 (5.0–8.5)	6.7 (4.4–9.0)	3.0 (2.0–4.0)
Non-Hispanic Asian	13.1 (10.6–15.6)	7.2 (6.1–8.2)	3.1 (2.0–4.1)	1.1 (0.6–1.7)	5.3 (4.3–6.4)	3.7 (2.9–4.6)	2.8 (2.0–3.5)	0.9 (0.5–1.3)
Non-Hispanic Black	19.9 (17.1–22.7)	6.3 (4.6–8.0)	6.0 (4.2–7.8)	1.2 (0.4–1.9)	18.5 (15.9–21.1)	6.3 (4.9–7.7)	6.7 (5.6–7.8)	1.8 (0.7–2.9)
Non-Hispanic White	45.7 (39.1–52.3)	74.2 (70.9–77.4)	80.8 (76.7–85.0)	92.9 (89.4–96.5)	62.8 (59.1–66.5)	81.2 (78.7–83.8)	80.8 (77.9–83.7)	91.8 (89.8–93.9)
Non-Hispanic Other	1.4 (1.0–1.8)	1.3 (0.9–1.6)	1.3 (0.8–1.7)	2.0 (1.2–2.7)	1.9 (1.3–2.4)	2.0 (1.5–2.5)	3.0 (2.4–3.6)	2.5 (1.5–3.5)
**Education level**								
Less than high school	12.1 (9.7–14.4)	7.3 (6.2–8.4)	8.6 (7.2–10.0)	10.0 (7.2–12.8)	9.0 (7.5–10.4)	6.0 (5.0–7.0)	8.2 (6.6–9.8)	11.1 (8.9–13.3)
High school diploma/GED	26.0 (24.2–27.9)	26.0 (24.3–27.7)	31.4 (28.4–34.4)	34.8 (30.0–39.5)	22.2 (20.4–23.9)	26.9 (25.3–28.5)	30.9 (28.1–33.8)	37.9 (35.0–40.8)
Some college/associate degree	22.9 (20.9–24.9)	25.5 (24.1–26.8)	26.9 (25.0–28.8)	31.2 (27.5–34.9)	28.3 (26.6–30.0)	31.3 (29.6–33.0)	31.8 (29.9–33.6)	31.9 (29.7–34.2)
Bachelor’s degree or higher	39.0 (36.6–41.3)	41.3 (39.0–43.6)	33.1 (29.4–36.7)	24.0 (18.5–29.5)	40.6 (38.1–43.1)	35.8 (33.3–38.3)	29.2 (25.4–32.9)	19.1 (16.9–21.2)
**Family income-to-poverty ratio, %**								
<100	13.5 (11.4–15.5)	5.0 (4.1–5.8)	7.7 (6.3–9.1)	10.4 (7.4–13.3)	10.8 (9.3–12.3)	5.2 (4.3–6.0)	10.0 (8.5–11.6)	10.2 (8.2–12.3)
100–199	18.4 (16.3–20.4)	12.2 (10.8–13.5)	15.5 (13.5–17.6)	20.0 (16.4–23.5)	16.9 (15.3–18.5)	12.1 (10.8–13.4)	17.0 (15.4–18.7)	21.1 (19.3–22.8)
200–299	14.6 (13.5–15.8)	12.1 (11.1–13.2)	16.1 (14.3–18.0)	18.1 (16.0–20.3)	15.4 (14.0–16.8)	14.8 (13.4–16.2)	18.0 (16.9–19.1)	20.1 (18.7–21.5)
300–399	11.5 (10.3–12.7)	12.6 (11.5–13.6)	14.3 (13.1–15.5)	15.2 (12.2–18.2)	11.9 (10.8–13.1)	15.0 (14.0–16.0)	15.3 (14.3–16.3)	16.6 (15.4–17.8)
400–499	9.2 (7.9–10.6)	11.7 (10.7–12.8)	12.1 (10.6–13.6)	12.2 (10.0–14.4)	10.5 (9.6–11.3)	14.2 (13.1–15.2)	12.5 (11.6–13.5)	13.1 (11.7–14.5)
≥500	32.8 (30.1–35.4)	46.4 (44.1–48.8)	34.2 (31.0–37.5)	24.1 (18.3–30.0)	34.5 (32.0–37.0)	38.8 (36.0–41.5)	27.1 (24.5–29.7)	18.9 (16.7–21.1)
**Body weight status**								
Underweight/normal weight	36.5 (34.8–38.3)	35.9 (34.3–37.4)	32.3 (29.8–34.8)	28.5 (25.8–31.3)	34.5 (32.7–36.4)	32.8 (32.2–34.3)	30.3 (28.5–32.1)	26.2 (24.5–27.8)
Overweight	33.7 (32.3–35.1)	35.1 (33.6–36.7)	33.3 (31.8–34.8)	30.4 (27.9–32.8)	35.0 (33.4–36.5)	32.6 (31.1–34.1)	31.5 (30.3–32.7)	32.4 (30.6–34.2)
Obese	26.3 (24.8–27.9)	26.2 (24.3–28.0)	31.3 (28.9–33.6)	38.1 (35.1–41.1)	28.5 (26.3–30.7)	32.6 (30.8–34.3)	36.0 (34.3–37.7)	39.0 (37.3–40.6)
Missing	3.5 (2.8–4.2)	2.8 (2.2–3.5)	3.1 (2.4–3.8)	3.0 (1.9–4.2)	2.0 (1.4–2.5)	2.1 (1.6–2.6)	2.2 (1.6–2.9)	2.4 (1.8–3.0)

Abbreviation: GED, general educational development.

a Not significant according to χ^2^ test (*P* > .05). All other values significant at *P* < .05 of characteristic differing according to county metropolitan status within region.

**Table 1b:** Demographic Characteristics of Adults, by Region and County Metropolitan Status, US South and West Regions, National Health Interview Survey, 2019–2022

Characteristic	South (n = 39,671), % (95% CI)				West (n = 27,345), % (95% CI)			
	Large central metro (n = 10,167)	Large fringe metro (n = 9,572)	Small/ medium metro (n = 13,108)	Nonmetro (n = 6,824)	Large central metro (n = 12,056)	Large fringe metro (n = 3,434)	Small/ medium metro (n = 9,291)	Nonmetro (n = 2,564)
**Age, y**								
18–44	52.0 (50.4–53.7)	45.2 (43.8–46.6)	43.7 (41.9–45.6)	36.4 (36.0–40.7)	50.4 (49.0–51.8)	49.0 (46.3–51.8)	47.2 (44.2–50.3)	40.9 (36.8–44.9)
45–64	30.5 (29.1–31.9)	33.8 (32.6–35.0)	32.4 (31.3–33.6)	34.5 (33.0–36.0)	31.1 (29.9–32.3)	30.9 (28.9–32.8)	30.2 (28.5–32.0)	34.5 (31.9–37.2)
65–74	10.4 (9.7–11.2)	12.6 (11.8–13.3)	13.9 (13.0–14.8)	15.8 (14.5–17.1)	10.6 (9.9–11.3)	11.7 (10.7–12.8)	13.8 (12.5–15.1)	15.3 (13.3–17.4)
≥75	7.0 (6.4–7.7)	8.4 (7.7–9.1)	10.0 (9.1–10.9)	11.3 (10.2–12.5)	7.9 (7.3–8.5)	8.4 (6.9–9.8)	8.7 (7.8–9.6)	9.3 (8.0–10.6)
**Sex**								
Female	52.3 (51.2–53.4)	51.2 (50.1–52.4)	53.7 (52.6–54.8)	53.4 (52.0–54.8)	49.8 (48.8–50.8)^a^	51.3 (49.2–53.4)^a^	51.1 (50.0–52.2)^a^	50.9 (48.1–53.7)^a^
Male	47.7 (45.6–48.8)	48.8 (47.6–49.9)	46.3 (45.2–47.4)	46.6 (45.2–48.0)	50.2 (49.2–51.2)^a^	48.7 (46.6–50.8)^a^	48.9 (47.8–50.0)^a^	49.1 (46.3–51.9)^a^
**Race and ethnicity**								
Hispanic	29.4 (24.9–33.9)	14.5 (12.2–16.7)	13.4 (8.4–18.4)	8.2 (1.5–14.9)	33.1 (29.8–36.4)	25.8 (20.0–31.6)	26.6 (20.2–32.9)	11.5 (6.6–13.3)
Non-Hispanic Asian	5.8 (4.9–6.7)	6.6 (5.3–7.9)	1.6 (1.3–2.0)	0.6 (0.4–0.8)	15.7 (13.4–17.9)	9.2 (7.2–11.3)	6.5 (3.4–9.7)	2.5 (0.1–4.9)
Non-Hispanic Black	22.7 (19.8–25.7)	18.3 (15.5–21.1)	18.8 (15.6–22.1)	15.4 (10.5–20.3)	5.7 (4.8–6.6)	5.4 (4.4–6.5)	2.2 (1.4–2.9)	0.5 (0.1–0.8)
Non-Hispanic White	40.2 (33.4–43.9)	58.6 (55.1–62.1)	63.9 (59.4–68.4)	71.6 (64.4–78.8)	42.6 (38.9–46.3)	56.1 (49.6–62.7)	60.6 (53.3–67.9)	72.0 (55.5–88.5)
Non-Hispanic Other	1.9 (1.5–2.3)	2.0 (1.5–2.4)	2.2 (1.5–3.0)	4.2 (1.9–6.4)	3.0 (2.5–3.4)	3.4 (2.6–4.2)	4.2 (3.0–5.4)	13.6 (0.0–29.9)
**Education level**								
Less than high school	13.2 (11.7–14.6)	9.4 (8.4–10.4)	12.7 (11.2–14.2)	19.3 (17.0–21.7)	12.2 (10.9–13.5)	10.1 (7.6–12.6)	12.5 (9.7–15.3)	13.1 (9.9–16.2)
High school diploma/GED	24.6 (22.9–26.4)	25.2 (23.6–26.8)	31.4 (29.8–33.0)	36.0 (34.1–37.9)	22.5 (20.9–24.0)	23.2 (21.5–25.0)	25.3 (23.0–27.6)	31.6 (28.9–34.3)
Some college/associate degree	26.3 (25.0–27.7)	29.4 (27.7–31.0)	30.6 (29.3–32.0)	28.9 (26.8–31.0)	28.2 (26.8–29.5)	32.3 (29.5–35.2)	34.9 (32.8–37.0)	34.7 (31.6–37.9)
Bachelor’s degree or higher	35.9 (33.1–38.7)	36.0 (33.4–38.6)	25.3 (23.5–27.1)	15.8 (13.9–17.7)	37.2 (34.5–39.9)	34.3 (30.8–37.9)	27.3 (24.3–30.3)	20.6 (15.4–25.8)
**Family income-to-poverty ratio, %**								
<100	12.2 (10.9–13.5)	7.0 (6.2–7.9)	12.9 (11.4–14.5)	17.3 (14.8–19.8)	8.9 (7.9–9.8)	7.0 (5.8–8.1)	9.9 (8.2–11.5)	12.8 (6.4–19.3)
100–199	19.4 (17.9–20.9)	14.7 (13.2–16.3)	21.8 (20.6–23.0)	26.7 (25.3–28.2)	16.9 (15.4–18.4)	14.5 (12.2–16.7)	19.0 (17.2–20.9)	20.1 (17.0–23.2)
200–299	17.0 (15.9–18.2)	15.6 (14.3–16.8)	17.5 (16.6–18.5)	19.8 (18.5–21.1)	15.2 (14.1–16.2)	16.1 (14.3–18.0)	17.8 (16.6–19.1)	19.2 (17.1–21.4)
300–399	12.0 (11.1–12.8)	13.9 (12.9–14.9)	13.6 (12.8–14.3)	12.6 (11.4–13.8)	12.5 (11.7–13.4)	13.8 (11.9–15.6)	14.1 (12.9–15.2)	13.4 (10.8–16.0)
400–499	9.9 (9.1–10.8)	12.0 (11.0–12.9)	11.1 (10.4–11.8)	9.5 (8.4–10.6)	9.7 (8.9–10.5)	11.2 (9.7–12.7)	11.6 (10.6–12.6)	9.9 (7.7–12.1)
≥500	29.5 (27.0–32.0)	36.8 (34.1–39.5)	23.1 (21.3–24.9)	14.1 (12.7–15.5)	36.8 (34.2–39.3)	37.4 (33.4–41.4)	27.6 (24.6–30.5)	24.5 (17.5–31.5)
**Body weight status**								
Underweight/normal weight	33.1 (31.7–34.6)	32.8 (31.5–34.1)	28.4 (27.2–29.5)	26.0 (24.3–27.6)	38.6 (37.2–40.0)	33.3 (30.9–35.6)	34.1 (32.1–36.1)	31.2 (25.7–36.7)
Overweight	33.2 (31.9–34.5)	33.2 (31.9–34.5)	32.7 (31.6–33.8)	31.0 (29.7–32.3)	33.6 (32.5–34.7)	34.8 (32.6–36.9)	33.4 (32.1–34.7)	31.7 (29.4–34.0)
Obese	31.1 (29.7–32.4)	31.8 (30.5–33.1)	36.5 (35.1–37.9)	40.9 (39.1–42.7)	25.8 (24.3–27.2)	30.1 (27.6–36.7)	30.4 (29.0–31.9)	35.1 (28.6–41.5)
Missing	2.6 (2.2–3.0)	2.2 (1.8–2.6)	2.4 (2.0–2.9)	2.1 (1.6–2.6)	2.0 (1.7–2.4)	1.8 (1.3–2.4)	2.0 (1.5–2.5)	2.0 (1.3–2.8)

Abbreviation: GED, general educational development.

a Not significant according to χ^2^ test (*P* > .05). All other values significant at *P* < .05 of characteristic differing according to county metropolitan status within region.

Unadjusted diabetes prevalence differed by metropolitan residence in the Northeast and South, with prevalence highest among adults residing in nonmetro counties and lowest among those in large fringe metro counties (Figure). Unadjusted diabetes prevalence among adults from nonmetro counties ranged from 9.0% (95% CI, 7.0%–11.1%) in the West to 14.8% (95% CI, 13.5%–16.1%) in the South.

**Figure:**
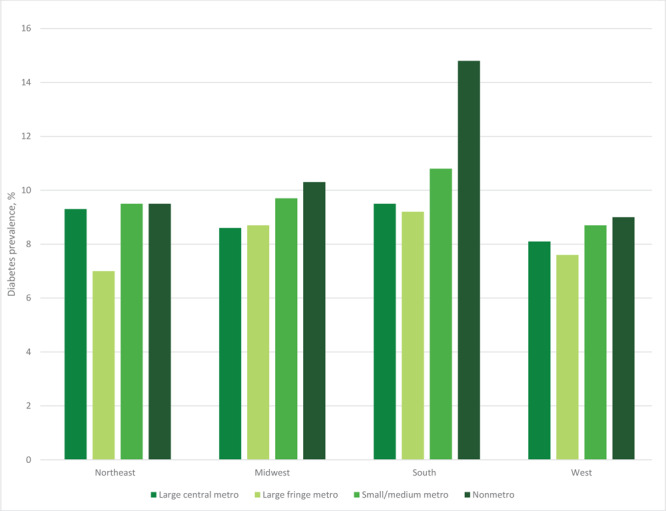
Unadjusted prevalence of self-reported diagnosed diabetes according to US census region and metropolitan status of county of residence, United States, 2019–2022.

**Table:** 

Census region	Metropolitan status of county of residence, prevalence (%)			
	Large central metro	Large fringe metro	Small/medium metro	Nonmetro
Northeast	9.3	7.0	9.5	9.5
Midwest	8.6	8.7	9.7	10.3
South	9.5	9.2	10.8	14.8
West	8.1	7.6	8.7	9.0

A significant interaction was detected between region and metropolitan residence in the logistic regression model adjusting for age, sex, and race and ethnicity (*P = .*01, Table 2). Compared with adults from large central metro counties, those from small/medium metro counties had significantly higher odds of diabetes in both the Midwest (OR = 1.24; 95% CI, 1.06–1.45) and South (OR = 1.15; 95% CI, 1.02–1.30). In the South region only, adults from nonmetro counties had significantly higher odds of diabetes compared with those from large central metro counties (OR = 1.62; 95% CI, 1.43–1.84). After further adjustment for socioeconomic status variables, the interaction between region and metropolitan residence remained significant (*P = .*01) and small/medium metro counties had significantly higher odds of diabetes only in the Northeast (OR = 1.16; 95% CI, 1.00–1.34). Nonmetro county residence remained significantly associated with diabetes in the South (OR = 1.30; 95% CI, 1.15–1.47). After further adjustment for body weight status, this interaction remained significant and only nonmetro county residence in the South remained significantly associated with diabetes (OR = 1.22; 95% CI, 1.07–1.39).

**Table 2:** Association Between County Metropolitan Status and Prevalence of Self-Reported Diagnosed Diabetes, by US Census Region, National Health Interview Survey, 2019–2022

Census region	Large fringe metro	Small/medium metro	Nonmetro
	Odds ratio^a^ (95% CI)		
**Model 1^b^**			
Northeast	0.84 (0.72–0.99)^c^	1.15 (0.99–1.34)	1.18 (0.95–1.45)
Midwest	1.04 (0.87–1.25)	1.24 (1.06–1.45)^c^	1.17 (0.97–1.40)
South	0.97 (0.86–1.11)	1.15 (1.02–1.30)^c^	1.62 (1.43–1.84)^c^
West	1.00 (0.84–1.19)	1.13 (0.98–1.30)	1.16 (0.90–1.48)
**Model 2^b^**			
Northeast	0.91 (0.78–1.07)	1.16 (1.00–1.34)^c^	1.05 (0.89–1.25)
Midwest	1.05 (0.88–1.26)	1.15 (0.99–1.34)	0.99 (0.82–1.20)
South	1.00 (0.88–1.13)	1.05 (0.94–1.18)	1.30 (1.15–1.47)^c^
West	1.00 (0.85–1.17)	1.06 (0.93–1.21)	1.00 (0.79–1.26)
**Model 3^b^**			
Northeast	0.90 (0.76–1.06)	1.10 (0.95–1.28)	0.94 (0.79–1.12)
Midwest	1.00 (0.83–1.20)	1.06 (0.92–1.23)	0.89 (0.74–1.07)
South	0.99 (0.87–1.12)	1.00 (0.89–1.13)	1.22 (1.07–1.39)^c^
West	0.95 (0.80–1.12)	1.04 (0.91–1.19)	0.94 (0.77–1.16)

a Odds ratios and confidence intervals shown for each model reflect parameterization of region and metropolitan status main effect coefficients and corresponding interaction terms. Estimates represent the association of metropolitan residence and diabetes prevalence within each region. Model 1 joint interaction, *P* = .002; model 2 joint interaction, *P* = .01; model 3 joint interaction, *P* = .008.

b Model 1 adjusted for age, sex, and race and ethnicity; model 2 adjusted for model 1 covariates plus income-to-poverty ratio and educational attainment; model 3 adjusted for model 2 covariates plus body weight status; reference group for each region is large central metro.

c *P* < .05.

## Discussion

The results of our study suggest that the association of metropolitan status with diabetes prevalence is not homogenous across the US. Rather, the highest unadjusted prevalence of diabetes was observed among adults residing in nonmetro counties in the South (14.8%). The odds of having diabetes were 62% higher among Southern nonmetro residents compared with those from large central metro counties after adjustment for age, sex, and race and ethnicity, and this association remained significant, though reduced, after further adjustment for income, education, and body weight status. By contrast, residence in nonmetro counties in other regions of the US was not associated with higher odds of diabetes. Higher odds of diabetes were also observed among residents of small and medium metro counties in the Northeast, Midwest, and South as compared with large metro counties within their respective regions, although these associations became nonsignificant after further adjustment for income, education, and body weight status.

Numerous disparities in health (15,16), health behaviors (6,17), socioeconomic status (18), and access to health care (19) have been reported among those living in rural areas. However, relatively fewer studies have examined how rural health disparities may differ across regions of the US. Although all rural, nonmetro counties in the US typically share characteristics such as lower population density and distance from large metropolitan areas, they may differ in terms of racial and ethnic distribution, socioeconomic status, the environment, and economy. For example, although nonmetro counties across every region have larger proportions of non-Hispanic White residents compared with large metro counties, Southern and Western nonmetro counties have smaller majorities of non-Hispanic White residents with greater proportions of Black residents in the South and Hispanic, Asian, and NH Other residents in the West (20). Furthermore, although rural–urban disparities in poverty and educational attainment are observed across all US regions, they manifest more severely in the rural South. Similar patterns in race and ethnicity, poverty, and educational attainment across region and metropolitan status were observed in our study. However, controlling for these variables in multivariable models did not fully explain the association of nonmetro county status with greater diabetes prevalence in the South. Regarding environment and economy, nonmetro counties can vary from those with tourist economies based on natural amenities such as mountains and lakes, to agricultural areas where cultivated fields or range land stretch for large distances, to places where mining or manufacturing is the key economic activity (19). These differences in environment and economy may further affect employment opportunities and commuting distances, access to health care, the retail food environment, and opportunities for physical activity (19). Unfortunately, exploring the potential effect of these environmental and economic contextual factors was not possible in this study because these data are not available in the NHIS data set.

This finding of elevated diabetes prevalence in the nonmetro South is consistent with research regarding diabetes mortality rates (10), diabetes incidence among the Veterans Administration patient population (12), and hospitalization rates following diabetes-related emergency department visits (11). Furthermore, the Southeastern region has long been designated as the “stroke belt” due to elevated stroke mortality rates observed since the middle of the 20th century, and stroke incidence has been observed to be particularly high among nonmetro areas in the South (21). Likewise, more recent research using Bayesian multilevel modeling of Behavioral Risk Factor Surveillance System data has also proposed a “diabetes belt” that occurs in the South (22). The factors contributing to the elevated prevalence of stroke and diabetes in the rural South are not entirely understood (21,22) but could include greater prevalence of risk factors such as lower socioeconomic status, obesity (3), poor diet (23), and insufficient physical activity (7). Although the association of diabetes with nonmetro county residence in the South was attenuated when we controlled for age, race and ethnicity, socioeconomic status, and body weight status, these factors did not entirely explain the association. Unfortunately, we were not able to assess whether physical activity or dietary quality explained the increased prevalence because data on these variables were not available for the entirety of the study period. However, in previous research by Barker et al regarding the “diabetes belt,” sociodemographic factors, body weight status, and sedentary lifestyle did not fully account for increased diabetes prevalence observed (22). Some literature also suggests that other unmeasured social factors such as discrimination and institutional racism could help explain the increased prevalence in the rural South, but information on these factors was also unavailable in our data (24). Finally, higher diabetes prevalence in the nonmetro South may also be linked to limited health insurance access among low-income populations, who are disproportionately concentrated there. As of May 2024, 7 of the 10 states that have not adopted Medicaid expansion under the Affordable Care Act to cover adults with incomes up to 138% of the poverty line are in the South census region (25). However, data on state of residence is unavailable in public use data, so we were unable to assess the potential impact of state Medicaid eligibility criteria on the results.

We also observed greater prevalence of diabetes among adults living in small and medium metro counties in the Midwest and South. However, our results suggest that this increased prevalence was largely explained by disparities in socioeconomic status, as these associations became nonsignificant when we controlled for income and education and further attenuated when we controlled for body weight status. Smaller cities in the Midwest and South, particularly those reliant on manufacturing, have been disrupted in recent decades by foreign trade and automation and have seen slower growth in employment and income compared with larger cities (26). We also observed a significant association of small and medium metro residence with diabetes in the Northeast after controlling for socioeconomic status, although the odds ratio remained of similar magnitude as in the previous model. This association may have been due to increased prevalence of risk factors such as body weight status, as the association became insignificant and attenuated after controlling for this variable.

Our study has several limitations. We relied on self-report of diabetes, which may be subject to misclassification; self-report also does not capture undiagnosed diabetes, which may occur more frequently among people without sufficient access to health care such as in nonmetro areas, although research suggests that diabetes screening rates are similar in urban and rural counties (27). Furthermore, we did not have adequate data on physical activity, dietary intake, or distance from health care resources, which could help elucidate potential mechanisms by which metropolitan residence could be associated with diabetes. Nonetheless, the large sample size allowed us to examine how the association of metropolitan residence with diabetes differs across US regions. In addition, our use of county metropolitan status as a proxy measure for rurality may limit the generalizability of the results since counties across metropolitan status categories may contain both urban and rural places and populations (28).

In conclusion, we found that the association of metropolitan residence with diabetes prevalence differs across regions of the US. These findings can help to guide efforts in areas where diabetes prevention and care resources may be better directed. Future research on rural–urban health disparities may consider examining how these disparities differ across US regions.
